# Supplementary material for: Neural circuit selective for fast but not slow dopamine increases in drug reward
Source: Nat Commun. 2023 Nov 8;14:6408. doi: 10.1038/s41467-023-41972-6 (PMC10632365; doi:10.1038/s41467-023-41972-6)
Supplement: Supplementary file 1 — Supplementary Information [file 41467_2023_41972_MOESM1_ESM.pdf]

Supplementary Information

Neural circuit selective for fast but not slow dopamine increases in drug reward

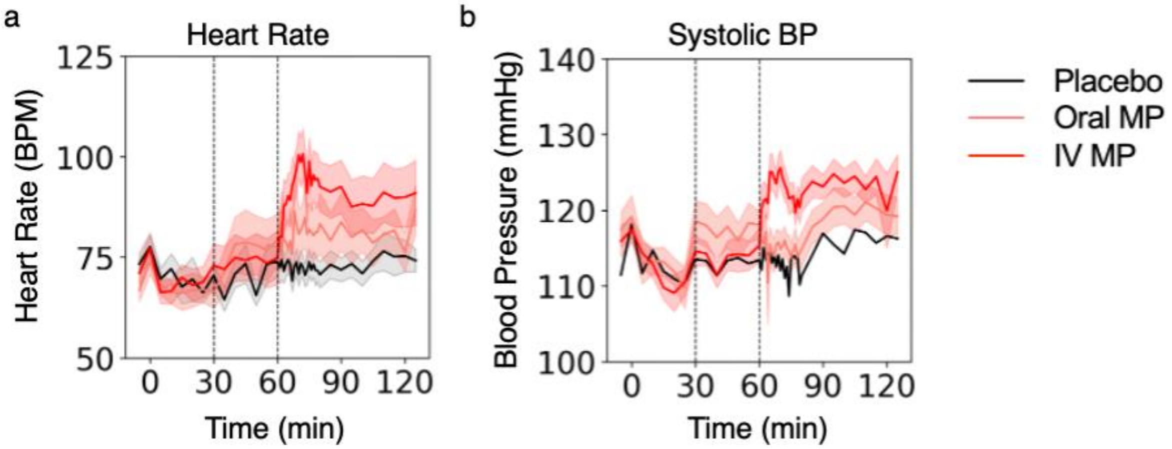

Supplementary Figure 1. Cardiovascular responses to methylphenidate (MP). The lines represent the mean of the 20 participants, and the shaded regions represent the standard error of the mean; the vertical dashed line at x=30 denotes the time of the raclopride injection, and the vertical dashed line at x=60 denotes the time of the IV MP or placebo injection. The black color denotes the placebo session; pink denotes the oral MP session; and red denotes the IV session.

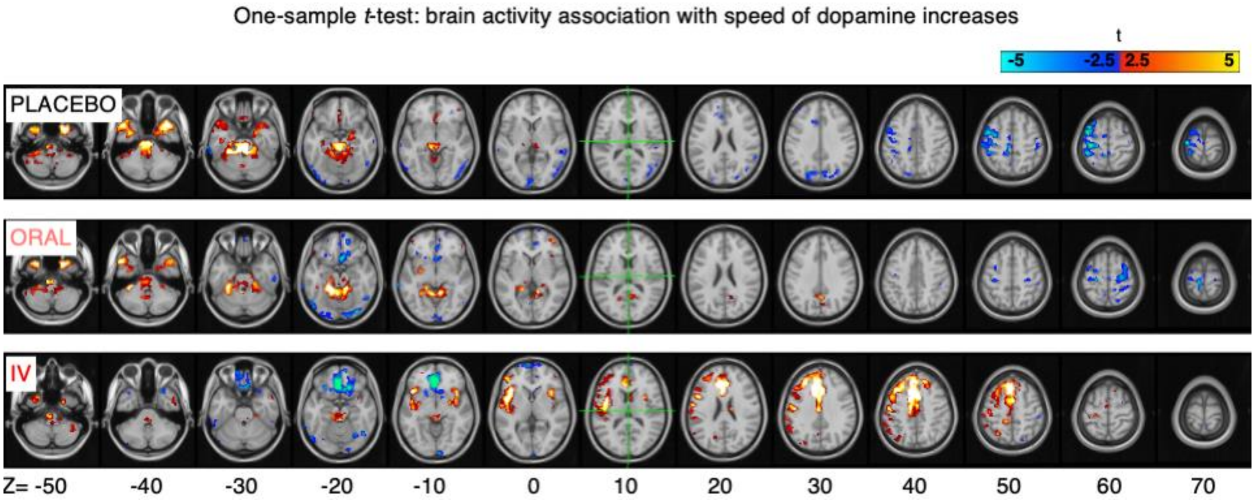

Supplementary Figure 2. One-sample t-tests (n=20). Whole brain analysis depicting the association of brain activity with speed of dopamine increases (i.e., the regressors shown in **Figure 2c** of the manuscript).

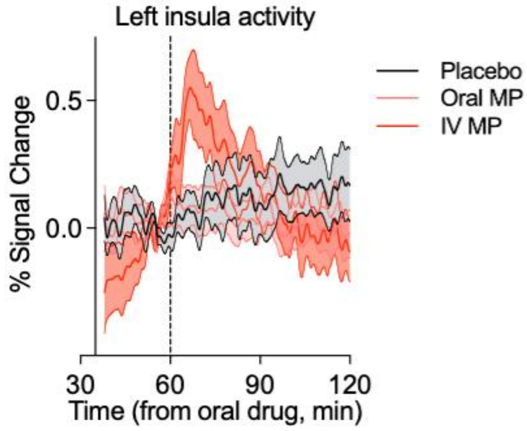

Supplementary Figure 3. The left insula cluster (from **Figure 2e** of the main manuscript) which was selectively activated to fast (IV) methylphenidate (MP). The lines represent the mean of the 20 participants, and the shaded regions represent the standard error of the mean; the vertical dashed line denotes the time of the IV injection. The black color denotes the placebo session; pink denotes the oral MP session; and red denotes the IV session.

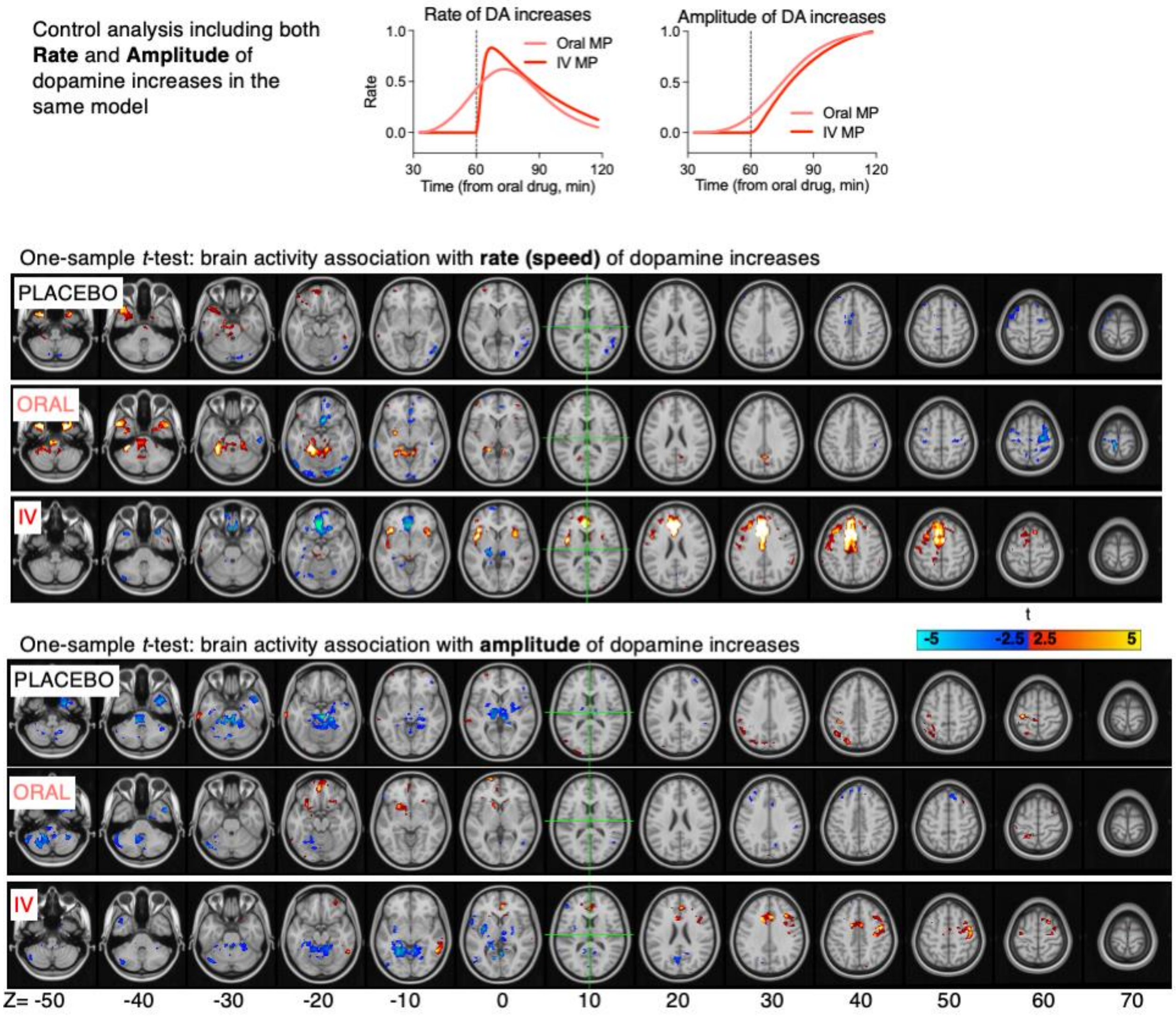

Supplementary Figure 4. One-sample t-tests: control analysis depicting the association of brain activity with *amplitude* of dopamine increases incorporated into the multiple regression model. Results for rate (speed) of dopamine increases remained nearly identical to those of the analyses presented in the main manuscript. In the top panel, the lines represent the mean of the 20 participants; the vertical dashed line denotes the time of the IV injection; the pink color denotes the oral MP session, and red denotes the IV session.

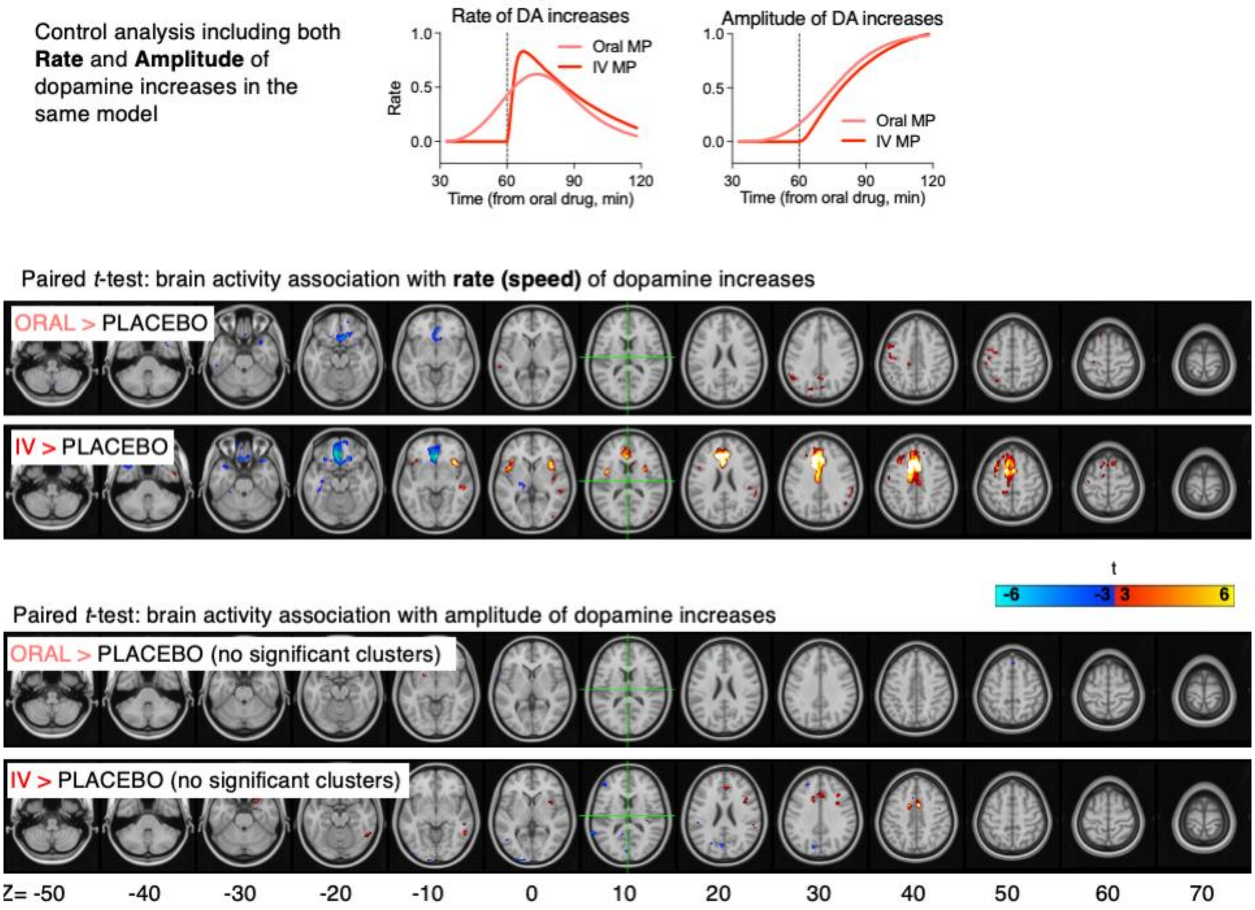

Supplementary Figure 5. Paired t-tests: control analysis depicting the association of brain activity with *amplitude* of dopamine increases incorporated into the multiple regression model. Results for rate (speed) of dopamine increases remained nearly identical to those of the analyses presented in the main manuscript (**Figure 2d,e**), whereas there were no significant clusters of brain activity in association with amplitude of dopamine increases in this model. In the top panel, the lines represent the mean of the 20 participants; the vertical dashed line denotes the time of the IV injection; the pink color denotes the oral MP session, and red denotes the IV session.

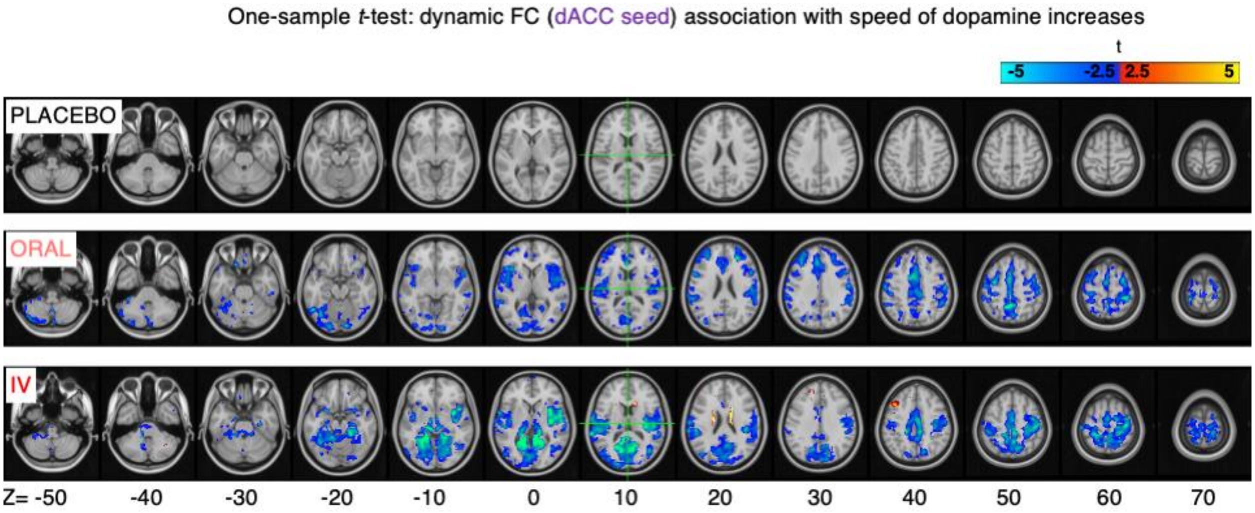

Supplementary Figure 6. Whole brain analysis depicting the association of dynamic brain functional connectivity (FC) with speed of dopamine increases (i.e., the regressors shown in **Figure 2c** of the manuscript); n=20. The seed region for functional connectivity was the significant dorsal anterior cingulate cortex (dACC) cluster from the primary analysis in the manuscript and shown in **Figure 2e**.

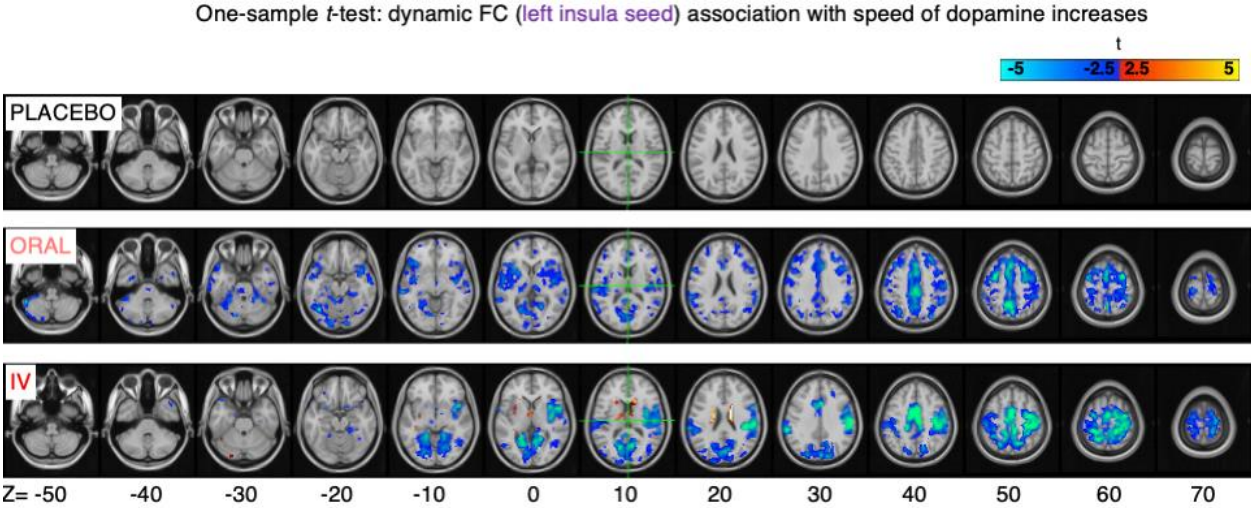

Supplementary Figure 7. Whole brain analysis depicting the association of dynamic brain functional connectivity (FC) with speed of dopamine increases (i.e., the regressors shown in **Figure 2c** of the manuscript); n=20. The seed region for functional connectivity was the significant left insula cluster from the primary analysis in the manuscript and shown in **Figure 2e**.

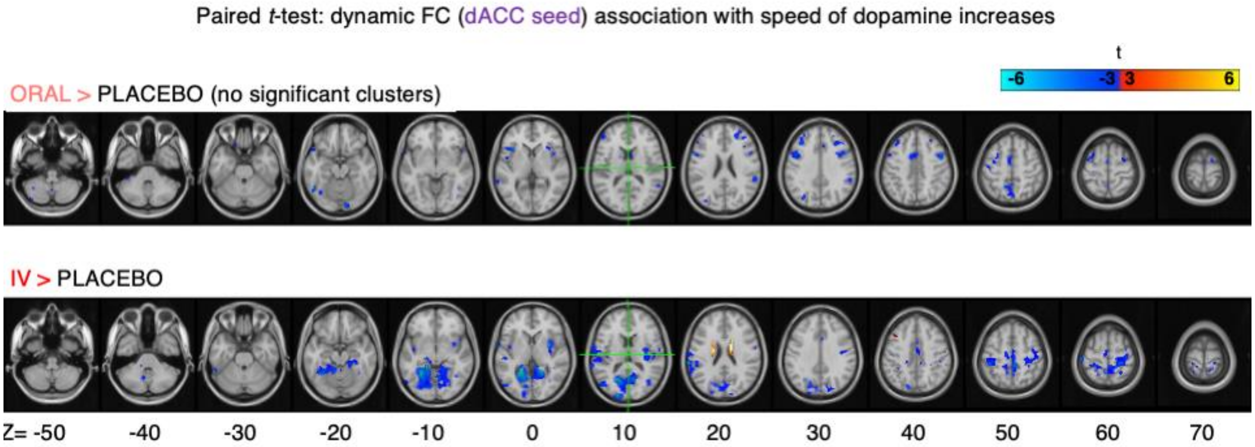

Supplementary Figure 8. Whole brain analysis depicting the association of dynamic brain functional connectivity (FC) with speed of dopamine increases (i.e., the regressors shown in **Figure 2c** of the manuscript); n=20. The seed region for functional connectivity was the significant dorsal anterior cingulate cortex (dACC) cluster from the primary analysis in the manuscript and shown in **Figure 2e, h, i**.

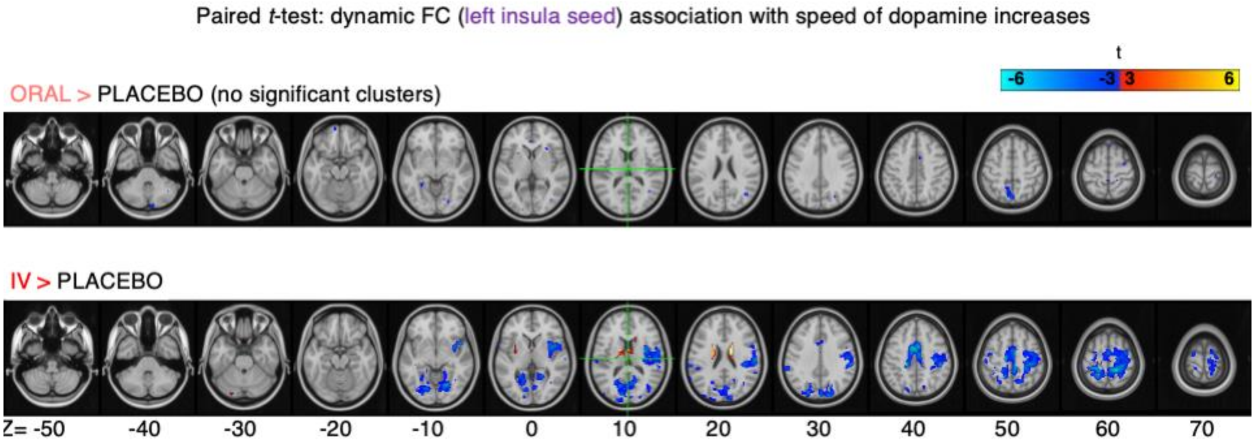

Supplementary Figure 9. Whole brain analysis depicting the association of dynamic brain functional connectivity (FC) with speed of dopamine increases (i.e., the regressors shown in **Figure 2c** of the manuscript); n=20. The seed region for functional connectivity was the significant left insula cluster from the primary analysis in the manuscript and shown in **Figure 2e**.

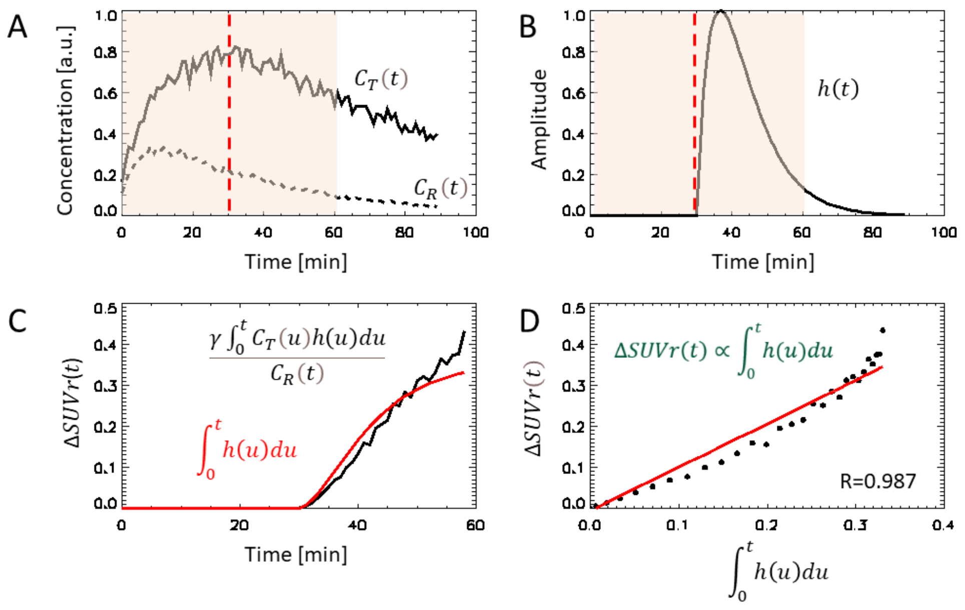

Supplementary Figure 10: Simulations demonstrating the similarity between the $\Delta SUVr$ method used in the current study to estimate dynamic dopamine increases, and the 'linear simplified reference region model' (LSSRM) method that has been used in prior studies. A) Time-varying concentrations of [11C]raclopride in the striatum, $C_T(t)$, and in the cerebellum, $C_R(t)$. B) A gamma variate function modeling the endogenous dopamine increases elicited by methylphenidate (MP), $h(t)$. C) Dynamics of SUVr changes, $\Delta SUVr(t)$, caused by MP-related increases in endogenous dopamine modeled with the LSSRM (black) and the approximation used in this study. D) Linear association between the exact and approximated LSSRM solutions. Normal random noise (5%) was added to $C_R(t)$ and $C_T(t)$. Dashed lines indicate the time of MP injection.

Supplementary Table 1. Demographic summary for the participants (n = 20 healthy adults).

**Age**	Mean ± SD	36.07 ± 9.80
**Sex**	n, Male/Female (%)	11/9 (55/45)
**BMI**	Mean ± SD	26.96 ± 2.66
**IQ**	Mean ± SD	108.7 ± 11.6
**Race**	n, White (%)	6 (30)
	n, Black/AA (%)	10 (50)
	n, Asian (%)	2 (10)
	n, Other (%)	2 (10)

Supplementary Table 2. Significant clusters from BOLD analysis: *activity changes in response to slow and fast dopamine increases* (corresponding to Figure 2d,e in the manuscript). Note: vmPFC = ventromedial prefrontal cortex; PLA = placebo; MNI = Montreal Neurological Institute

	Region	pFWE	pFDR	k	peak T	peak Z	Coordinates (MNI)
**SLOW DOPAMINE INCREASES (ORAL > PLA)**							
POSITIVE	(no significant clusters)						
NEGATIVE	vmPFC	< .001	< .001	160	6.55	4.63	[9 38 -12]
					5.71	4.26	[12 23 -15]
					5.58	4.20	[21 27 -12
**FAST DOPAMINE INCREASES (IV > PLA)**							
POSITIVE	dACC	< .001	< .001	2366	12.92	6.40	[-9 12 36]
					11.65	6.14	[-9 26 30]
					9.29	5.56	[6 15 42]
	Insula Left	< .001	< .001	204	7.06	4.83	[-33 21 6]
					5.91	4.35	[-42 18 -6]
					5.81	4.31	[-36 0 12]
NEGATIVE	vmPFC	< .001	< .001	554	8.30	5.26	[6 30 -9]
					7.45	4.97	[-3 33 -12]
					6.21	4.48	[21 24 -15]

Supplementary Table 3. Significant clusters from analysis: *connectivity changes (seed region: dACC) in response to slow and fast dopamine increases* (corresponding to Figure 3 and Supplementary Figure 8). Note: PLA = placebo; MNI = Montreal Neurological Institute

	Region	pFWE	pFDR	k	peak T	peak Z	Coordinates (MNI)
**SLOW DOPAMINE INCREASES (ORAL > PLA)**							
POSITIVE	(no significant clusters)						
NEGATIVE	(no significant clusters)						
**FAST DOPAMINE INCREASES (IV > PLA)**							
POSITIVE	Dorsal Caudate Right	0.035	0.014	63	7.42	5.03	[18 0 27]
					6.93	4.84	[18 15 21]
					5.69	4.29	[15 -15 24]
	Dorsal Caudate Left	0.039	0.014	61	6.99	4.86	[-18 9 21]
					6.24	4.55	[-18 -15 24]
NEGATIVE	Calcarine Left	< .001	< .001	895	6.62	4.71	[-24 -57 -3]
					6.44	4.63	[-12 -39 -6]
					5.95	4.42	[-18 -48 3]
	V1 Left	0.013	0.027	80	5.56	4.23	[-12 -93 15]
					4.80	3.84	[-12 -99 6]
					4.28	3.53	[-21 -87 18]
	Somatomotor Right	< .001	< .001	173	5.22	4.06	[39 -21 69]
					4.83	3.85	[9 -30 51]
					4.61	3.73	[18 -45 69]
	Temporo-Parietal Left	0.017	0.027	75	5.16	4.03	[-63 -33 24]
					4.65	3.75	[-48 -30 18]
					4.42	3.62	[-66 -39 12]

Supplementary Table 4. Significant clusters from analysis: *connectivity changes (seed region: left insula) in response to slow and fast dopamine increases* (corresponding to Supplementary Figure 9). Note: SMA = supplementary motor area; PLA = placebo; MNI = Montreal Neurological Institute

**SLOW DOPAMINE INCREASES (ORAL > PLA)**							
POSITIVE	(no significant clusters)						
NEGATIVE	(no significant clusters)						
**FAST DOPAMINE INCREASES (IV > PLA)**							
POSITIVE	Dorsal Caudate Bilateral	< .001	< .001	204	6.37	4.60	[15 -6 24]
					6.27	4.56	[-18 3 27]
					6.21	4.53	[-18 -6 27]
NEGATIVE	Somatomotor/SMA	< .001	< .001	2143	6.32	4.58	[-9 -21 51]
					6.19	4.52	[-15 -30 42]
					6.12	4.49	[9 -12 51]
	Calcarine Right	0.022	0.032	77	5.71	4.31	[21 -69 -3]
					4.05	3.40	[9 -66 -3]
					3.68	3.16	[9 -78 -6]
	Calcarine Left	< .001	< .001	497	5.69	4.30	[-21 -69 9]
					5.58	4.24	[27 -69 27]
					4.98	3.94	[-15 -81 30]
