# Supplementary material for: Neural circuit selective for fast but not slow dopamine increases in drug reward
Source: Nat Commun. 2023 Nov 8;14:6408. doi: 10.1038/s41467-023-41972-6 (PMC10632365; doi:10.1038/s41467-023-41972-6)
Supplement: Supplementary file 3 — Reporting Summary [file 41467_2023_41972_MOESM3_ESM.pdf]

nature portfolio

Corresponding author(s): Peter Manza and Nora D. Volkow

Last updated by author(s): September 3, 2023

Reporting Summary

Nature Portfolio wishes to improve the reproducibility of the work that we publish. This form provides structure for consistency and transparency in reporting. For further information on Nature Portfolio policies, see our Editorial Policies and the Editorial Policy Checklist.

Statistics

For all statistical analyses, confirm that the following items are present in the figure legend, table legend, main text, or Methods section.

n/a	Confirmed	
☐	☒	The exact sample size (*n*) for each experimental group/condition, given as a discrete number and unit of measurement
☐	☒	A statement on whether measurements were taken from distinct samples or whether the same sample was measured repeatedly
☐	☒	The statistical test(s) used AND whether they are one- or two-sided<br>*Only common tests should be described solely by name; describe more complex techniques in the Methods section.*
☐	☒	A description of all covariates tested
☐	☒	A description of any assumptions or corrections, such as tests of normality and adjustment for multiple comparisons
☐	☒	A full description of the statistical parameters including central tendency (e.g. means) or other basic estimates (e.g. regression coefficient) AND variation (e.g. standard deviation) or associated estimates of uncertainty (e.g. confidence intervals)
☐	☒	For null hypothesis testing, the test statistic (e.g. *F*, *t*, *r*) with confidence intervals, effect sizes, degrees of freedom and *P* value noted<br>*Give P values as exact values whenever suitable.*
☒	☐	For Bayesian analysis, information on the choice of priors and Markov chain Monte Carlo settings
☒	☐	For hierarchical and complex designs, identification of the appropriate level for tests and full reporting of outcomes
☒	☐	Estimates of effect sizes (e.g. Cohen's *d*, Pearson's *r*), indicating how they were calculated

Our web collection on statistics for biologists contains articles on many of the points above.

Software and code

Policy information about availability of computer code

Data collection	High rating prompts were displayed on a projector using a program (E-Prime Version 3.0) designed to minimize visual stimulation.
Data analysis	The minimal preprocessing pipelines of the Human Connectome Project (HCP) were used for image processing. Specifically, FreeSurfer 5.3.0 (http://surfer.nmr.mgh.harvard.edu) was used for automatic segmentation of anatomical MRI scans into cortical and subcortical gray matter ROIs3 Then, for the EPI images, the FSL Software Library (version 5.0; http://www.fmrib.ox.ac.uk/fsl) was used for rigid body realignment, field map processing, co-registration to the anatomical T1 image, and spatial normalization to MNI space.<br>We further processed the EPI images for resting fMRI analysis, including: regression of white matter, CSF, and global signals using custom MATLAB code; and 5 mm full-width at half-maximum spatial smoothing, using FSL.<br><br>Cardiovascular responses were analyzed using repeated measures drug condition x time ANOVA, with the aov function in R v 3.6.3.<br>PET dynamic dopamine increases were computed using custom code in the interactive data language (IDL).<br>Whole-brain voxelwise multiple regression analysis of fMRI data were conducted in SPM12.<br>Time series regression analysis of dynamic functional connectivity with high ratings using the 'dyn' and 'lm' packages in R v 3.6.3.

For manuscripts utilizing custom algorithms or software that are central to the research but not yet described in published literature, software must be made available to editors and reviewers. We strongly encourage code deposition in a community repository (e.g. GitHub). See the Nature Portfolio guidelines for submitting code & software for further information.

Data

Policy information about availability of data

All manuscripts must include a data availability statement. This statement should provide the following information, where applicable:

- Accession codes, unique identifiers, or web links for publicly available datasets
- A description of any restrictions on data availability
- For clinical datasets or third party data, please ensure that the statement adheres to our policy

Summary data will be made available in a public repository upon publication.

Human research participants

Policy information about studies involving human research participants and Sex and Gender in Research.

Reporting on sex and gender	Sample included 9 females and 11 males.
Population characteristics	Twenty healthy individuals (36.1±9.6 years old) participated; further details are in Supplementary Table 1.
Recruitment	Participants were recruited through referrals from the NIH Volunteer Office, the Patient Recruitment and Public Liaison (PRPL) Office, ResearchMatch.org, by word of mouth, and through Institutional Review Board (IRB)-approved advertisements.
Ethics oversight	The study was approved by the IRB at the National Institutes of Health Intramural Research Program.

Field-specific reporting

Please select the one below that is the best fit for your research. If you are not sure, read the appropriate sections before making your selection.

[x] Life sciences   [ ] Behavioural & social sciences   [ ] Ecological, evolutionary & environmental sciences

For a reference copy of the document with all sections, see nature.com/documents/nr-reporting-summary-flat.pdf

Life sciences study design

All studies must disclose on these points even when the disclosure is negative.

Sample size	Sample size was determined based on prior PET studies in our lab and others demonstrating adequate power to detect large effect sizes. (see methods section, 'Participants' subheading.)
Data exclusions	Five subjects could not complete the study due to technical failures in data acquisition and therefore were not included in final data analysis.
Replication	Due to the highly costly nature of this research it was not possible for us to conduct replication experiments.
Randomization	The session order was randomized across participants by the NIH clinical center pharmacy (true randomization, blocked every 6 subjects).
Blinding	Participants and researchers were blind to medication (methylphenidate and placebo) and route of administration (oral and intravenous).

Reporting for specific materials, systems and methods

We require information from authors about some types of materials, experimental systems and methods used in many studies. Here, indicate whether each material, system or method listed is relevant to your study. If you are not sure if a list item applies to your research, read the appropriate section before selecting a response.

Materials & experimental systems

n/a	Involved in the study
☒	☐ Antibodies
☒	☐ Eukaryotic cell lines
☒	☐ Palaeontology and archaeology
☒	☐ Animals and other organisms
☐	☒ Clinical data
☒	☐ Dual use research of concern

Methods

n/a	Involved in the study
☒	☐ ChIP-seq
☒	☐ Flow cytometry
☐	☒ MRI-based neuroimaging

Clinical data

Policy information about clinical studies

All manuscripts should comply with the ICMJEguidelines for publication of clinical research and a completedCONSORT checklist must be included with all submissions.

Clinical trial registration	NCT03326245
Study protocol	https://clinicalstudies.info.nih.gov/ProtocolDetails.aspx?id=2017-AA-0178
Data collection	Data were collected at the NIH Clinical Center in Bethesda, Maryland from January 2018 to September 2021
Outcomes	Primary imaging measures were speed of dopamine increases (estimated by dynamic PET modeling) and their association with brain activity (estimated by fMRI signal activity); for more details see 'PET image analysis: estimation of dynamic dopamine increases to oral and IV MP' and 'fMRI image analysis: activity changes in response to slow and fast dopamine increases' paragraphs in the methods section. Secondary outcomes were the fMRI association with behavior ('high' ratings).

Magnetic resonance imaging

Experimental design

Design type	Resting state
Design specifications	One 90-minute continuous session
Behavioral performance measures	High ratings were assessed throughout the scan on a scale of 1-10. High rating prompts occurred every 5 min from the onset of oral methylphenidate administration; then, at the onset of intravenous methylphenidate administration, prompts occurred every minute for 20 min. Then, prompts occurred every 5 min until the end of scanning.

Acquisition

Imaging type(s)	Functional MRI and Positron Emission Tomography
Field strength	3T
Sequence & imaging parameters	FMRI was conducted with a single-shot echo planar imaging (EPI) sequence (TE/TR = 30/3000 ms, FOV = 192 × 192 mm, in-plane resolution = 3 × 3 mm, 1800 volumes, 36 slices/volume, slice thickness = 4mm)
Area of acquisition	Whole-brain

Diffusion MRI ☐ Used ☒ Not used

Preprocessing

Preprocessing software	The minimal preprocessing pipelines of the Human Connectome Project (HCP)38 were used for image processing. Specifically, FreeSurfer 5.3.0 (http://surfer.nmr.mgh.harvard.edu) was used for automatic segmentation of anatomical MRI scans into cortical and subcortical gray matter ROIs39. Then, for the EPI images, the FSL Software Library (version 5.0; http://www.fmrib.ox.ac.uk/fsl)40 was used for rigid body realignment, field map processing, co-registration to the anatomical T1 image, and spatial normalization to MNI space.<br>We further processed the EPI images for resting fMRI analysis, including: regression of white matter, CSF, and global signals using custom MATLAB code; and 5 mm full-width at half-maximum spatial smoothing, using FSL. For dynamic resting connectivity analysis only, we also bandpass filtered the fMRI data in the .01 - .1 hz frequency range.
Normalization	Non-linear normalization in FSL using FNIRT
Normalization template	MNI305 template normalization in SPM

Noise and artifact removal: We further processed the EPI images for resting fMRI analysis, including: regression of white matter, CSF, and global signals using custom MATLAB code; and 5 mm full-width at half-maximum spatial smoothing, using FSL. For dynamic resting connectivity analysis only, we also bandpass filtered the fMRI data in the .01 - .1 hz frequency range.

Volume censoring: Data were motion-corrected but not censored due to the need to correlate the fMRI time-series with the PET time series

Statistical modeling & inference

Model type and settings: First level: Multiple Regression Model
Second level: paired t-tests (whole-brain)

Effect(s) tested: N/A

Specify type of analysis: [x] Whole brain [ ] ROI-based [ ] Both

Statistic type for inference (See Eklund et al. 2016): For all whole-brain group level analyses, the significance threshold was set at voxelwise $p < .001$ uncorrected, with a cluster-forming threshold of $p < .05$ false discovery rate (FDR)-corrected, and a minimum cluster size of $k > 50$, in line with current reporting guidelines.

Correction: FDR Correction, see prior response.

Models & analysis

n/a	Involved in the study
[ ]	[x] Functional and/or effective connectivity
[x]	[ ] Graph analysis
[x]	[ ] Multivariate modeling or predictive analysis

Functional and/or effective connectivity: Pearson correlation
